# Tapered Waist Tensile Specimens for Evaluating Butt Fusion Joints of Polyethylene Pipes—Part 1: Development

**DOI:** 10.3390/polym14061187

**Published:** 2022-03-16

**Authors:** Sunwoo Kim, Taemin Eom, Wonjae Lee, Sunwoong Choi

**Affiliations:** 1 Department of Polymer Science and Engineering, Hannam University, Daejeon 34054, Korea; swkpado@krict.re.kr (S.K.); xoals2342@gmail.com (T.E.); dnjswo3983@naver.com (W.L.); 2 Chemical Materials Solutions Center, Korea Research Institute of Chemical Technology, Daejeon 34114, Korea

**Keywords:** polyethylene (PE) pipes, tapered waist tensile (TWT), waisted tensile (WT), butt fusion (BF) joints, fracture energy

## Abstract

The structural integrity of butt fusion (BF) joints in thermoplastic pressure piping systems is critical to their long-term safe use. The tapered waist tensile (TWT) specimen was developed to alleviate issues associated with ISO 13953 waisted tensile (WT) specimen for evaluating BF joints. Experimental and finite element analyses were performed to obtain optimum TWT specimen designs for the BF joint destructive test. For TWT specimens, depending on the pipe size, the displacement at onset necking was reduced by 30~100%, and the tested BF area increased by 60~80% compared to the WT specimen. In addition, the transverse specimen deflection was lower thus providing better experimental stability. Furthermore, it showed the same BF displacement at the maximum force local to the BF bead, indicating that the tapered waist geometry provides equivalent deformation constraint and BF failure mode designed for the BF joint in the WT specimens. Therefore, TWT specimens offer simplicity, adaptability, stability, and accuracy in specimen preparation, testing, and analysis compared to WT specimens.

## 1. Introduction

In creating a thermoplastic piping system for the conveyance of fluid, pipes are joined together, and the structural integrity of the joints produced is essential for ensuring their safety and reliability during their designed lifetimes. With the structural requirement of the joint met, polyethylene (PE) pipes are being used in a wide range of demanding applications across the water, gas, oil, and energy industries including nuclear [1]. Among several available jointing methods for thermoplastic pipes, thermal butt fusion (BF) [2] and electrofusion (EF) [3] jointing are popularly used, and their performance has proven dependable over decades of piping system operations. BF is one of the most frequently used in the PE piping system, and more stringent joint assessment requirements are considered as their applications widen into higher pressures and larger diameters pipes. Various specifications and standards for fusion procedure, long-term life design, performance, and installation procedures are utilized to meet such requirements, and new ones are made. In addition, several international, regional, and ad hoc standards are available that evaluate the BF joints by the destructive and non-destructive methods.

Destructive methods range from static tensile, bending, tensile creep, fatigue, and high-speed tensile tests. The static tensile method is most popularly used, and they include waisted tensile (WT) [4,5], dogbone tensile [6], and low-temperature tensile (LTT) [7] specimens. All have different geometries and contain BF joints at the center perpendicular to the specimen axis. A WT specimen maintains the BF bead, whereas dogbone can be tested either with a bead or without the bead. On the other hand, the low-temperature tensile (LTT) test is performed with the bead removed. WT specimen is designed to provide stress concentration at the waisted BF joint, while the reduced section in the LTT specimen is shaped by a large radius. In addition, WT and dogbone specimens are tested at room temperature, while −80 °C is used for the LTT test. Pull speeds of 5, 50, and 200 mm/min are applied, for WT, dogbone, and LTT specimens, respectively.

Using dogbone specimens, earlier workers determined the conditions for achieving good BF joints of polyethylene materials of different melt flow indexes [8] and of different grade polyethylene, polybutene-1, and polypropylene [9]. It was also determined that with the bead removed, it provided a clearer picture of the joint performance [9].

Tensile creep test [10] demonstrated that BF joints of large diameter and thick wall PE pipes, produced by the single low pressure (SLP) and duel low pressure (DLP) [11], had slow crack growth (SCG), starting at the stress concentration developed between the bead and the parent pipe and the crack growth occurring into the parent pipe [12]. It was also reported that BF made by the SLP procedure performed better than DLP.

The lifetime ranking of PE pipe BF joints by the SCG using the Pennsylvania notched test (PENT) [13,14] and the full notch creep test (FNCT) [15,16] have been reported. Using FNCT on thick wall BF joints produced by SLP, DLP, and SHP (single high pressure) procedures all demonstrated to satisfy the designed lifetime requirements. However, it was noted that the lifetime was less with SHP but not significantly. More recently, SCG times in BF joint were determined for different PENT-rated PE4710 pipes used for nuclear power applications [17]. The method showed the SLP procedure for BF jointing gave longer SCG failure times than the SHP method [18].

The long-term biaxial loading of BF joint using the plane strain grooved tensile (PSGT) specimens [19,20], removed from the 630 SDR 11 and 914 SDR 17.6 pipe BF joints, showed a comparable stress–rupture result to that of the sustained internal pressure pipe test [21]. Hence, this has provided an alternate means to stress–rupture test BF joint of large diameter pipes.

Stress controlled fatigue test on BF joints was reported. It showed that the BF bead reduced the low-cycle tensile fatigue lifetime and had a negligible effect on the medium and high-cycle lifetimes [22]; however, low and medium cycle regions were affected when tested in bending mode [23]. In addition, a deflection controlled fully reversed cantilever bending fatigue test was applied to PE4710 4” SDR 11 BF joints and showed better performance with unimodal than the bimodal resin [24]. A high-speed tensile impact test on BF joints using a tensile impact speed of 152 mm/min is often used to correlate to the indications from the non-destructive examination for determining between good and bad joint, based on the failure mode [25,26].

Evaluation of a BF joint using the bending mode is available through the technological bend test of DVS 2203-5 [27] and guided strip bend test of ASTM F3186 [28]. Bend angle (or ram displacement) and resulting deformation and fracture of the BF joints are used to assess the condition of the joints. More recently, Wermelinger proposed a modified strip bend test that evaluates BF joints with ease, quickness, and good accuracy [26].

All of the BF joint destructive tests described above was based on the tensile coupon test in which the coupons are removed from different locations in the BF joint. Studies of BF joint integrity using the whole pipe tensile test have also been reported. More recently, a whole pipe axial tensile test using hydrostatic pressure was developed and demonstrated its effectiveness by comparing it to the mechanical axial tension test result of the whole pipe BF joint [29]. Tensile creep tests on whole pipe BF joints using hydraulic jack [30], and hydrostatic pressure (HAT) [31,32] have also been reported. Both tests were made to test the BF joint with factors like residual stresses and fusion strength variation around the joint cross-section, otherwise not considered in the coupon creep tests. HAT has been shown to produce comparable results to the whole pipe mechanical creep test [21,31] and has the advantage of testing whole pipe BF joint of different sizes using already available hydrostatic pressure testing equipment.

Comparison of waisted tensile (WT) specimen to other specimens was discussed [30,33], and one of the advantages is that it allows tensile tests to be performed on BF joints from thick-wall pipes. In addition, a recent international round-robin on the performance of several BF joint test specimens has demonstrated that the WT test is one of the methods that can better discriminate between good and bad PE pipe BF joints [26].

The BF joint strength or fracture energy value is measured in the WT test. The joint strength is then compared to the pipe strength, and the “weld factor” (ratio between joint strength and pipe strength) is obtained [34,35]. The “weld factor” value determines between good versus the flawed joint. However, a shortcoming of this method is that joint fractures can occur in some flawed BF joints after achieving the same joint strength as no-flaw BF joints. Thus, a personal judgment is made by observing the fracture mode on the fracture surface to determine the state of the BF joint integrity. This makes the method subjective and can lead to erroneous conclusions of the BF joint quality. In the context of published studies of BF joint integrity, fracture energy is more frequently reported than strength [36], as fracture energy also takes into account the energy spent on fracturing the joint after reaching the maximum load. The quantitative fracture energy is estimated from the total area under the load–displacement curve. However, traction holes in the unreduced parts of the WT specimen for accommodating loading rods deform upon loading and become a significant added source of unnecessary specimen displacement included in the BF joint fracture energy calculation. In addition, in some pipe sizes, for a given standard dimensional ratio (SDR–ratio between pipe diameter and thickness), the number of WT specimens produced from the BF joint decreases with increasing pipe diameter. Hence, for 225 SDR 11 to 914 SDR 11 WT specimens, the % total area of the BF joint tested is between 19.5% and 23.1%. Therefore, the reliability of the test results in representing the entire BF joint can be questioned. In addition, the size, shape, and traction holes in the WT specimen can make the specimen machining to dimensions more demanding. Recently, work to optimize the WT specimen dimensions has been carried out [37]. However, although the modified WT specimen dimensions were changed to create ductile failures in the no-flaw BF joint and the parent pipe, it still retained the use of loading pins.

In this work, a tapered waist tensile (TWT) test specimen for testing the BF joint is developed that eliminated the use of loading pins. In addition, by redesigning the shape of the “waist” in the form of a tapered waist fusion zone notch, a single specimen width is made to cover all pipe sizes and pipe thicknesses. As a result, the specimens are shorter and simpler in shape with no traction holes, thus making the specimen machining simpler. In addition, a simple push-in tensile grip design is described, making the tensile test more stable and simpler. Experimental and numerical analyses are performed on TWT and WT specimens of high-density PE BF joints to compare their merits. Therefore, TWT specimens offer simplicity, adaptability, stability, and accuracy in specimen preparation, testing, and analysis compared to WT specimens.

## 2. Materials and Methods

### 2.1. Material

Materials were received as PE100 (HE3490-LS, Borouge, Abu Dhabi, UAE) black compound extruded pipes of 110 SDR 9*, 225 SDR 11, 315 SDR 11, and 450 SDR 9 size from a local pipe extruder (Cosmo I & D, Sejong, Korea). The material manufacturer declared tensile yield strength, fracture elongation, and modulus of elasticity were 25 MPa, >600%, and 1.1 GPa, as measured by ISO 527-2. The density and melt flow index of the PE resin were 960 kg/m^3^ (ISO 1183-12, Method 1) and 0.25 g/10 min (ISO 1133), respectively (https://borouge.com/IndustrySolution/PDF%20Files/DataSheetBB2581.pdf, assessed on 4 February 2022).

For example, a 110 SDR 9 pipe size refers to a pipe with 110 mm nominal outside diameter (*d_n_*) and a standard dimensional ratio (SDR–ratio between pipe diameter and thickness) of 9. Therefore, the nominal pipe wall thickness (*t*) was 12.2 mm.

### 2.2. Butt Fusion (BF) Jointing

BF joints containing no flaws were produced on 110 SDR 9, 225 SDR 11, 315 SDR 11, and 450 SDR 11 pipes. BF joints from the former three pipe sizes were made using a T-315 BF jointing machine (Georg Fischer, Schaffhausen, Switzerland), and 450 SDR 11 was produced using a BT216 butt fusion machine (WMS, Seoul, Korea), according to the BF procedure of ISO 21307. For all pipe sizes, a single low-pressure (SLP) procedure using 1.5 bar was followed with the heating plate set at 230 °C.

All butt-fused pipes were left at ambient temperature for 24 h before machining into TWT and WT specimens.

### 2.3. TWT and WT Specimen Preparation

**Figure 1:**
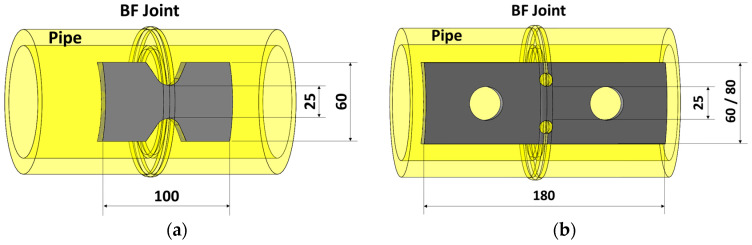
Diagram of relative positions of Type A specimens (unit: mm): (**a**) TWT; (**b**) WT.

Tapered waist tensile (TWT) and waisted tensile (WT) specimens were cut from the butt-fused pipe along the pipe axial, as shown in Figure 1. The BF bead is centered on each specimen and machined to dimensions on a Simplex-2 CNC milling machine (Hawcheon, Gwangju, Korea). The final shapes and dimensions of TWT and WT specimens made from 110 SDR 9, 225 SDR 11, 315 SDR 11, and 450 SDR 9 pipe BF joints and the maximum number of TWT and WT specimens possible from each pipe size are given in Section 3.1.1. All specimens were conditioned at 23 °C, 50% RH for at least 24 h prior to the tensile test.

### 2.4. TWT and WT Test

**Figure 2:**
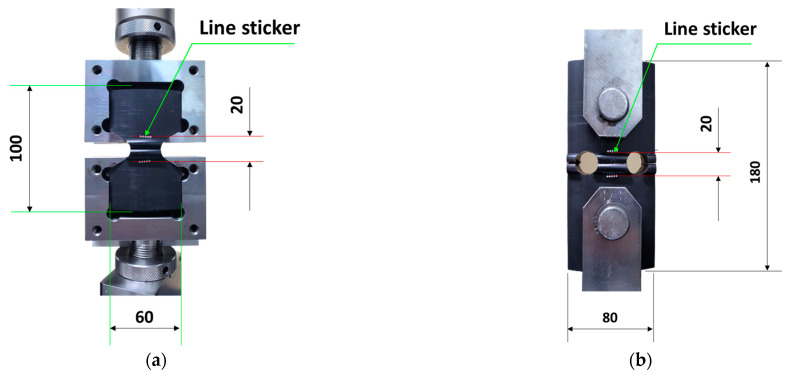
Grip-specimen assembly showing the line sticker gage location for optical measurement (unit: mm): (**a**) TWT test; (**b**) WT test.

TWT and WT specimens were tensile tested using a universal testing machine (AGS-X, Shimadzu, Kyoto, Japan). A 50 kN load cell and a dual-camera video extensometer (TRViewX 800D, Shimadzu, Kyoto, Japan) with up to 800 mm field of view measurement capability were employed. For the TWT specimen, a specially designed universal TWT push-in grip (see Figure 2a) was used. Traction holes were used with WT specimens to accommodate the loading pins for tensile testing (Figure 2b). The 5 mm/min displacement rate was used for TWT and WT specimens. Specimen displacement was measured in two ways. One used the grip to grip (machine crosshead) separation distance. And the other by the video extensometer with a gage length set at ±10 mm from the center of the bead width for all pipe sizes (Figure 2). All specimens were tested to failure, and the load–displacement curves were obtained for further analysis to determine the BF joint fracture energy. Tensile tests were carried out in the same environment as conditioning.

## 3. Results and Discussion

### 3.1. TWT Specimen Development

#### 3.1.1. Specimen Geometry and Dimensions

**Figure 3:**
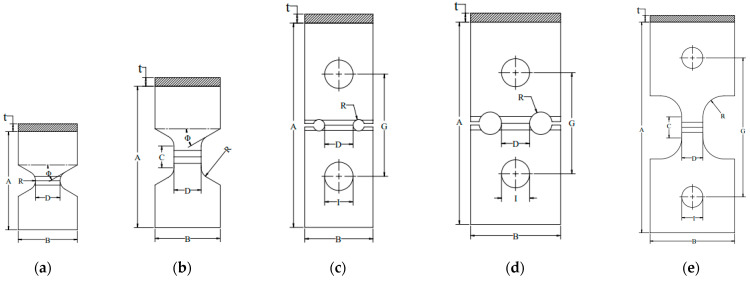
Shape and relative dimensions: (**a**) Type A TWT specimen (*t* < 25 mm); (**b**) Type B TWT (*t* ≥ 25 mm); (**c**) Type A WT specimen (*t* < 25 mm), $d_{n}$ ≤ 160 mm; (**d**) Type A WT (*t* < 25 mm), $d_{n}$ ≤ 160 mm; (**e**) Type B WT (*t* ≥ 25 mm).

The geometry and dimensions of the proposed TWT specimens with a BF bead at the center are given in Figure 3a,b, and Table 1. Similarly, Figure 3c–e, and Table 2 show the shape and dimensions of the WT specimens per ISO 13953. The characteristic design of the TWT specimen has a tapered waist cut in angles to provide tapered support for the tensile load. Two TWT specimen types A (Figure 3a) and B (Figure 3b), cover the BF tensile test for all pipe sizes. In comparison, WT specimens require three types (two Type A (Figure 3c,d) and a Type B (Figure 3e)). The TWT specimen is smaller and shorter than the WT specimen due to the absence of traction holes on the specimen, as shown in Figure 3. However, the width of the BF joint tested is the same in both specimens (*D* = 25 mm in Figure 3, Table 1 and Table 2). In addition, while the width of the WT specimen (dimension *B* in Table 2) increases with pipe dimensions, the width is fixed at 60 mm for TWT specimens for all pipe sizes. Hence, with the TWT specimen, the BF joint area tested is larger by 60%~80% than the WT specimen for pipes having greater than 160 mm nominal diameter, as shown in Table 3. The absence of traction holes in TWT specimens also provides simplicity of specimen machining, ease of testing, and experimental stability. At the same time minimizes the specimen tensile displacement contribution from places other than the BF area.

#### 3.1.2. Tapered Support Angle Grip Design

**Figure 4:**
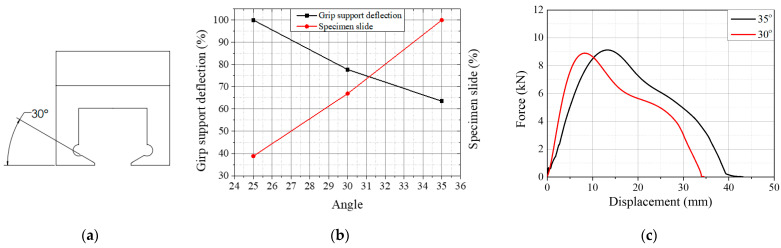
(**a**) Tapered support angle of push-in grip for TWT specimens; (**b**) finite element analysis result; (**c**) experimental load–displacement curves with 30° and 35° grip support angle.

In the TWT specimen, the tensile displacement from the specimen gripping is minimized by using a tapered support angle (*Φ* in Figure 3) in the newly designed push-in grip that supports reaction to the tensile load shown in Figure 4a.

The finite element result of the gripping is shown in Figure 4b for the support angle between 25° and 35° [38]. The grip support angle of 35° provides significant specimen slipping while the support angle deflection is the smallest under the tensile load. And vice versa with a 25° angle. That is, the larger the grip support angle, the more is the specimen slips but smaller the grip deflection under the tensile load. Therefore, a grip support angle of 30° was chosen to provide an optimum design for resistance against specimen slippage and gripping instability.

Figure 4c illustrates the experimental load–displacement curves of TWT specimens with grip support angles 30° and 35°. The load–displacement behavior is similar, but the 35° grip support angle exhibits a larger displacement at maximum load by about 55% than at 30°.

**Figure 5:**
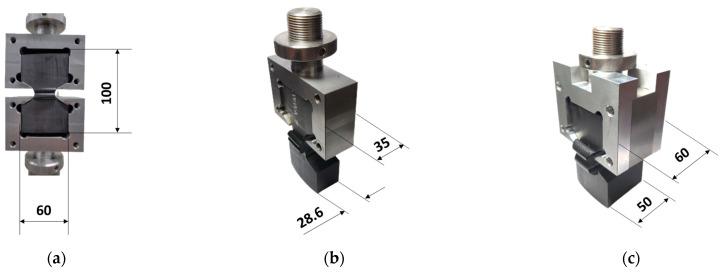
(**a**) Assembly of push-in grip and TWT specimen; (**b**) example of 315 SDR 11 (single-grip assembly for *t* ≤ 30 mm specimens); (**c**) example of 450 SDR 9 (multi-layering of grip assembly for testing thicker specimens of *t* > 30 mm) (unit: mm).

A photograph of the newly designed push-in tensile grip and TWT specimen assembly is shown in Figure 5a. Since the specimen width is the same independent of the pipe size, one push-in grip was made to fit all specimens with wall thickness ≤ 315 SDR 11 (Figure 5b). Multiple push-in grips can be assembled together for add-on thickness for thicker specimens, as illustrated in Figure 5c.

#### 3.1.3. Experimental Development of TWT Specimen

**Figure 6:**
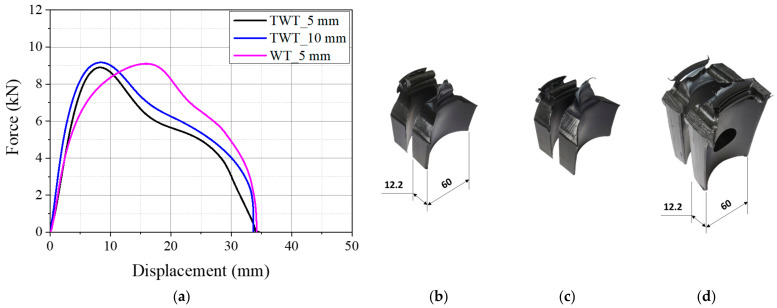
Results of 110 SDR 9 (Type A specimens) pipe BF joint (unit: mm): (**a**) load–displacement curves; (**b**) TWT_5 mm; (**c**) TWT_10 mm; (**d**) WT_5 mm fusion zone notch radius.

**Figure 7:**
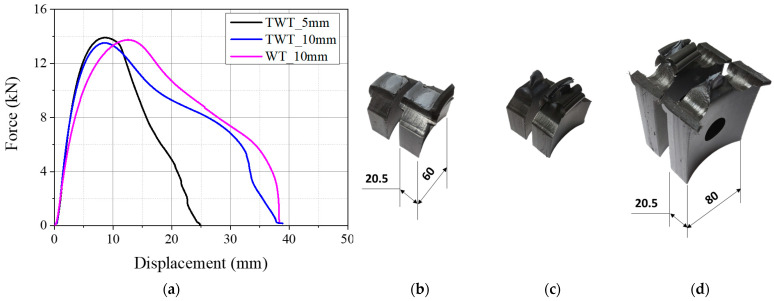
Results of 225 SDR 11 (Type A specimens) pipe BF joint (unit: mm): (**a**) load–displacement curves; (**b**) TWT_5 mm; (**c**) TWT_10 mm; (**d**) WT_10 mm fusion zone notch radius.

The fusion zone notch radius of the Type A TWT specimen was decided by tensile testing 5 mm and 10 mm radius notches. The load–displacement curves obtained at 5 mm/min crosshead speed are given in Figure 6a and Figure 7a for 110 SDR 9 and 225 SDR 11 TWT specimens, respectively. The corresponding failed specimens are presented in Figure 6b,c and Figure 7b,c. For the 110 SDR 9 TWT specimen, 5 and 10 mm notch radius show practically the same result (Figure 6a and Table 4). In addition, the maximum load is similar to the WT specimen (5 mm notch radius); however, the corresponding displacement is reduced by 52% with TWT specimens, as expected (Table 4). The failure mode is completely ductile and occurs after full necking displacements in all specimens (Figure 6b–d). These results indicate that a 5 or 10 mm fusion zone notch radius is suitable for use with the Type A 110 SDR 9 TWT specimen.

The Type A, 225 SDR 11 TWT specimen with a 5 and 10 mm radius fusion zone notch exhibits different load–displacement behavior as given in Figure 7a. Slightly higher maximum load and lower fracture displacement are shown for a 5 mm notch radius (Table 5). These may be partly due to the rate effect, where a 5 mm radius notch provided approximately two times higher nominal strain rate than a 10 mm notch radius at the BF zone. On the other hand, the 10 mm notch radius gives a similar load–displacement curve to the WT specimen (10 mm notch radius) but smaller specimen displacement by about 30% at the maximum load (Table 5). The corresponding failure mode is a mixed-mode failure with lower ductility for a 5 mm notch radius (Figure 7b). In contrast, similar complete ductile failure is seen with a 10 mm notch radius TWT (Figure 7c) and WT (Figure 7d) specimens. Hence for the Type A, 225 SDR 11 TWT specimen, a fusion zone notch radius of 10 mm is suitable.

Type B TWT and WT specimens are illustrated in Figure 3b,e. The TWT specimen had a shorter reduced parallel side (dimension *C* in Table 1 and Table 2). In addition, the grip width of the specimen end (dimension *B*) was 60 mm, whereas 100 mm width was used with the WT specimen. The length of the reduced parallel side of the Type B TWT specimen was decided by evaluating the parallel side length of 0, 10, and 20 mm on 315 SDR 11 (*t* = 28.6 mm) and 450 SDR 9 (*t* = 50 mm) sizes. The 0 mm parallel side length makes the same shape as the Type A specimen with a 10 mm fusion zone notch radius (dimension *R* in Figure 3a,b, and Table 1).

**Figure 8:**
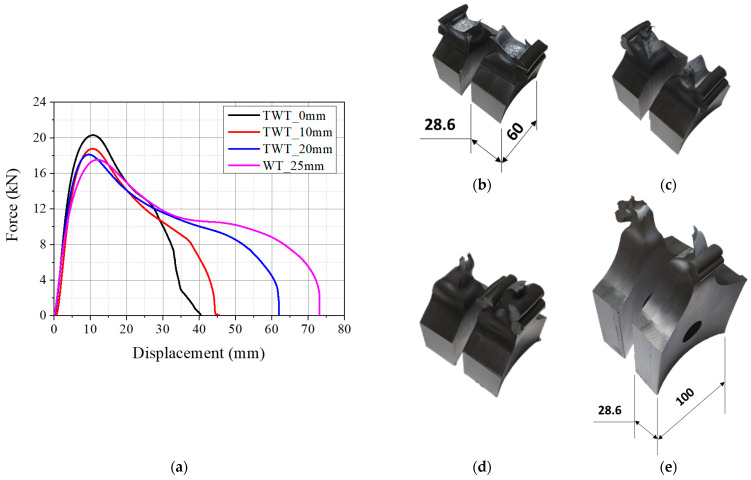
Results of 315 SDR 11 (Type B specimens) pipe BF joint (unit: mm): (**a**) load–displacement curves; (**b**) TWT)_0 mm; (**c**) TWT_10 mm; (**d**) TWT_20 mm; (**e**) WT_25 mm reduced parallel side length.

**Figure 9:**
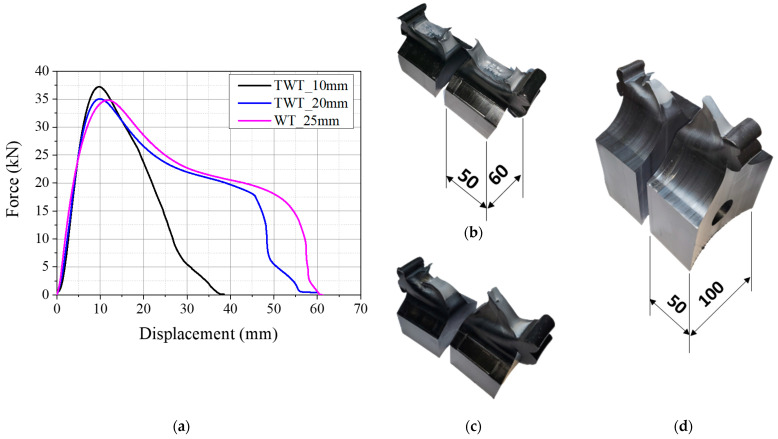
Results of 450 SDR 9 (Type B specimens) pipe BF joint (unit: mm): (**a**) load–displacement curves; (**b**) TWT_10 mm; (**c**) TWT_20 mm; (**d**) WT_25 mm reduced parallel side length.

The load–displacement curves of TWT specimens with 0, 10, and 20 mm parallel side lengths are given in Figure 8a and Figure 9a for 315 SDR 11 and 450 SDR 9 sizes, respectively. The corresponding modes of TWT specimen failure are also presented in Figure 8 and Figure 9. All load–displacement curves were obtained at a 5 mm/min crosshead speed.

For the 315 SDR 11 TWT specimen, the maximum load is seen to decrease with the parallel side length increase from 0, 10, and 20 mm. The displacement at maximum load remains about the same (Table 6), and the total displacement increases with increasing length (Figure 8a). The 20 mm parallel side length TWT compares best with the WT (25 mm) specimen in load–displacement behavior (Figure 8a and Table 6) and failure mode (Figure 8d,e). The 10 mm length also exhibited ductile yielding failure (Figure 8c), while the 0 mm length specimen was shown to have much reduced ductility at failure (Figure 8b).

The results show that the Type A TWT specimen (i.e., Type B with 0 mm length) is unsuitable for the specimen thickness larger than 25 mm (e.g., 315 SDR 11 and 450 SDR 9), and the Type B specimen with a parallel side length of 10 or 20 mm radius needs to be utilized.

Figure 9 illustrates the load–displacement curve and the failure appearance of TWT specimens taken from the 450 SDR 9 pipe BF joints. The 0 mm parallel side length is not tested as the failure occurred with lesser ductility when made from the 315 SDR 11 BF joint (Figure 8a,b). The maximum force decreases going from 10 to 20 mm parallel side. However, the corresponding specimen displacement remained the same. In addition, the total displacement increases with increasing length. The 20 mm parallel side TWT specimen compares best with the WT (25 mm) in load–displacement behavior and failure mode (Figure 9c,d). Although the displacement at maximum load is still smaller by about 30% than the WT specimen (Table 7). Ten millimeters length also exhibited ductile yielding failure; however, with much-reduced ductility, as shown in Figure 9b. The results show that a parallel side length of 20 mm is most suitable for Type B TWT specimens.

Therefore, based on the experimental results given above, a fusion zone notch radius of 10 mm (Type A, Figure 3a) and the parallel side length of 20 mm (Type B, Figure 3b) is most suitable to use for TWT specimens with a wall thickness of ≤25 mm and >25mm, respectively. These and other dimensions of Type A and Type B TWT specimens are given in Table 1.

#### 3.1.4. Test Speed Determination

**Figure 10:**
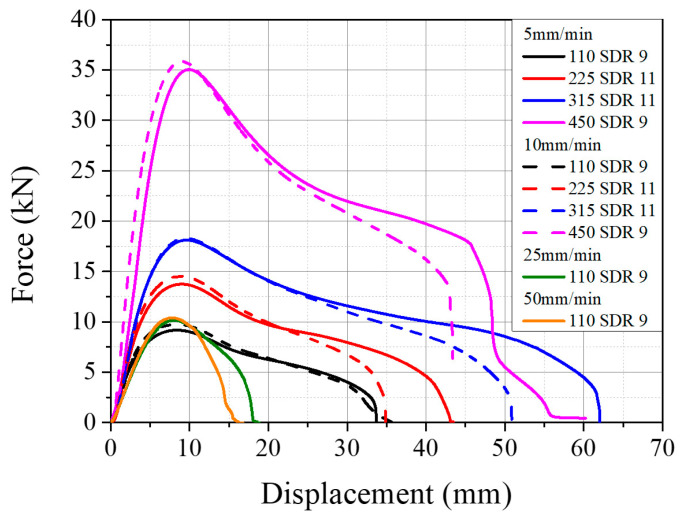
Effect of crosshead speed on load–displacement behavior of TWT specimens.

The effect of crosshead speed was investigated to determine the optimum test speed for the TWT specimen. Type A (110 SDR 9, 225 SDR 11) with fusion zone notch radius of 10 mm, and Type B (315 SDR 11, 450 SDR 9) having 20 mm parallel side length specimens were tested. Two crosshead speeds were used, 5 mm/min, and 10 mm/min. First, 5 mm/min was chosen as this speed is used in testing WT specimens per ISO 13953. Next, 10 mm/min was tested to see if the test time could be reduced. The higher speeds were not considered as 25 mm/min, and 50 mm/min noticeably reduced the elongation of the 110 SDR 9 TWT specimen, and much of the post-necking behavior differed compared to the 5 and 10 mm/min speed shown in Figure 6. However, the load–displacement curves of all sized TWT specimens are similar at 5 and 10 mm/min test speeds (Figure 10). In addition, although some reduced elongation with 10 compared to 5 mm/min speed, most of the post-necking behavior was retained to be similar at both speeds. Therefore, for the TWT dimensions given in Table 1, test speeds of 5 or 10 mm/min are suitable.

### 3.2. FE Analysis of TWT Specimen

**Figure 11:**
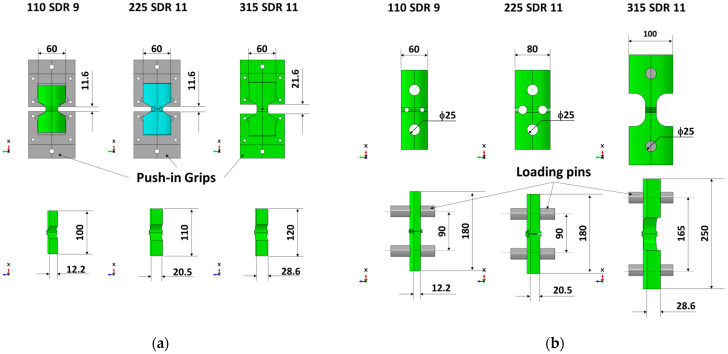
Specimen and grip assembly dimensions for FE analysis (unit: mm): (**a**) TWT; (**b**) WT.

**Figure 12:**
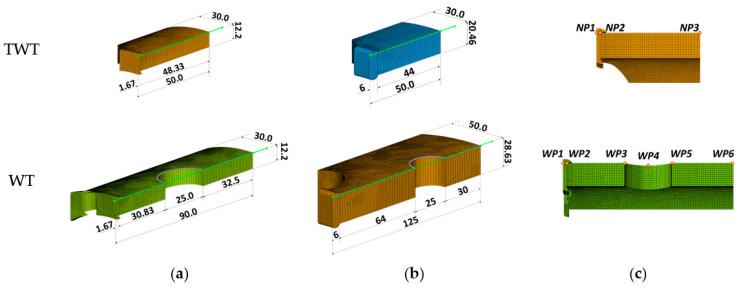
1/4 Finite element models and data collection lines along the longitudinal direction from the center of the BF joint to the tip of the specimen (unit: mm): (**a**) Type A model (110 SDR 9); (**b**) Type B model (315 SDR 11); (**c**) points of interest.

To better understand the deformation behavior of TWT specimens, a finite element (FE) analysis was performed using commercial finite element software ABAQUS. WT specimens are also analyzed for comparison. The specimens were modeled using a four-node linear tetrahedral element (C3D4 in ABAQUS) as illustrated in Figure 11 and Figure 12.

**Figure 13:**
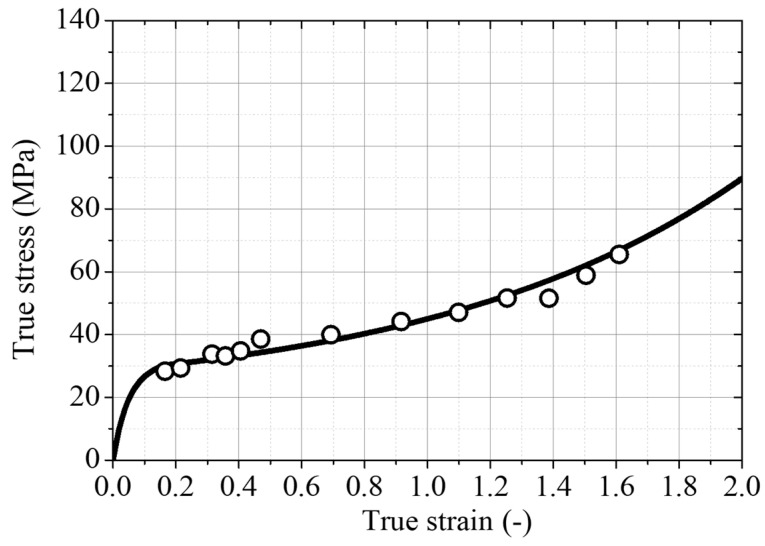
True stress–strain curve of PE 100 pipe grade resin.

The true stress–strain data of PE100 utilized for the FE analysis is shown in Figure 13. The true stress–strain data up to the onset necking was estimated from the nominal stress–strain data using Equations (1) and (2). An exponent value of *n* = 1.25 was chosen as it gave an optimal curve passing through the true stress and strain data points (open circles) obtained from the real-time experimental measurement of the cross-sectional area and the length change during the tensile deformation, as shown in Figure 13. The independent elastic constants (i.e., *E* = 1.1 GPa, *σ_y_* = 18.5 MPa, *ε_y_* = 0.03037, and *ν* = 0.45) were used to describe the elastic part. The plastic part after the yield strength was tabulated from the fitted true stress–strain curve.

(1) $$\sigma_{true}=\{\begin{array}{c} \sigma_{nom}\left(1+\varepsilon_{nom}\right),\;\;\;\;\;\;\;\;\;\sigma_{nom}\leq \sigma_{y}\\ \sigma_{y}\left(1+{\left(\varepsilon_{nom}-\varepsilon_{y}\right)}^{n}\right),\;\;\;\;\;\sigma_{nom}>\sigma_{y} \end{array}$$

(2) εtrue=∫l0ldl/l=ln(ll0)=ln(1+εnom)

where *σ_nom_*, *ε_nom_*, *σ_true_*, *ε_true,_ σ_y,_* *ε_y_*, and *n* are the nominal stress and strain, true stress and strain, true yield stress and strain, and plastic exponent, respectively. *l_0_* is the initial gauge length set at 50 mm, and *l* is the distance between gauge marks at any time.

The true stress–strain data of PE100 was further converted into appropriate elastic-plastic data for FE analysis using ABAQUS/Standard. The plastic strain is obtained by subtracting the elastic strain from the total strain, as shown in Equations (3) and (4).

(3) $$\varepsilon_{total}=\varepsilon_{true}=\varepsilon_{e}+\varepsilon_{p}$$

(4) $$\varepsilon_{p}=ln\left(1+\varepsilon_{nom}\right)-ln\left(1+\sigma_{y}/E\right),\;\varepsilon_{nom}\geq \varepsilon_{y}$$

where *ε_total_*, *ε_e_*, *ε_p_*, and *E* are the total strain, elastic strain, plastic strain, and elastic modulus, respectively.

**Figure 14:**
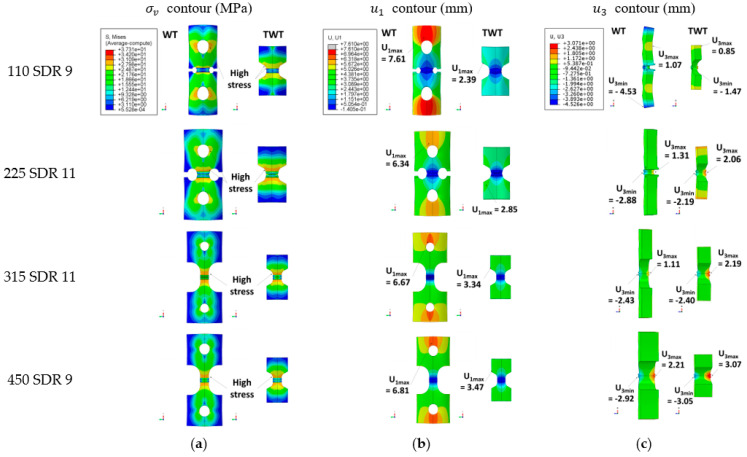
FE analysis results at *F_max_*: (**a**) $\sigma_{v}$; (**b**) $u_{1}$; (**c**) $u_{3}$ contours.

**Figure 15:**
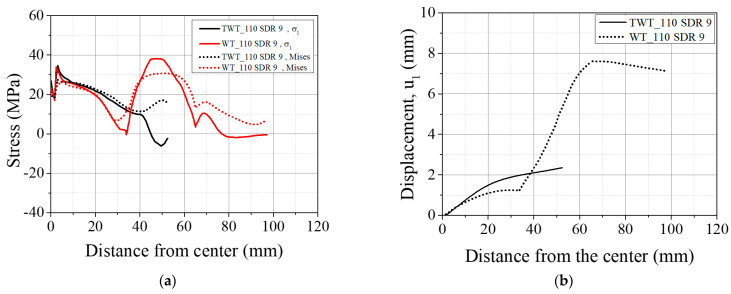
FEA results of Type A, 110 SDR 9 TWT and WT specimens at *F_max_*: (**a**) $\sigma_{v}$ and $\sigma_{1}$; (**b**) $u_{1}$.

**Figure 16:**
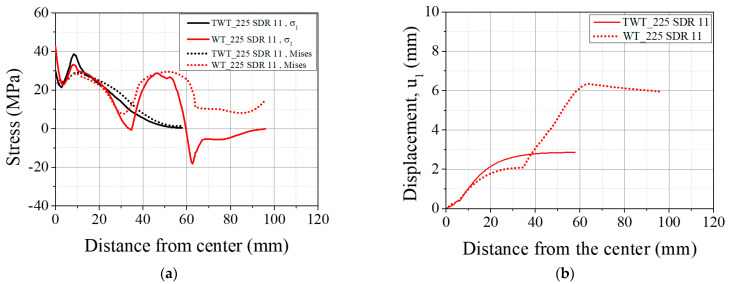
FEA results of Type A, 225 SDR 11 TWT and WT specimens at *F_max_*: (**a**) $\sigma_{v}$ and $\sigma_{1}$; (**b**) $u_{1}$.

**Figure 17:**
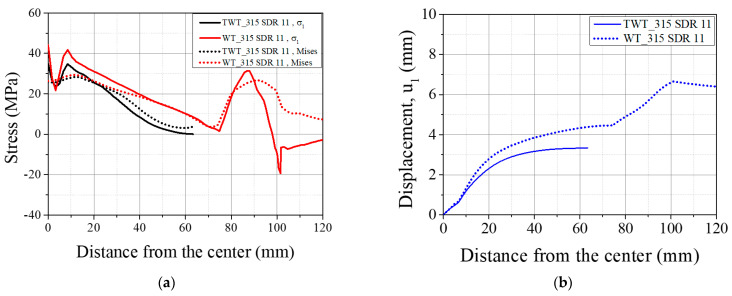
FEA results of Type B, 315 SDR 11 TWT and WT specimens at *F_max_*: (**a**) $\sigma_{v}$ and $\sigma_{1}$; (**b**) $u_{1}$.

**Figure 18:**
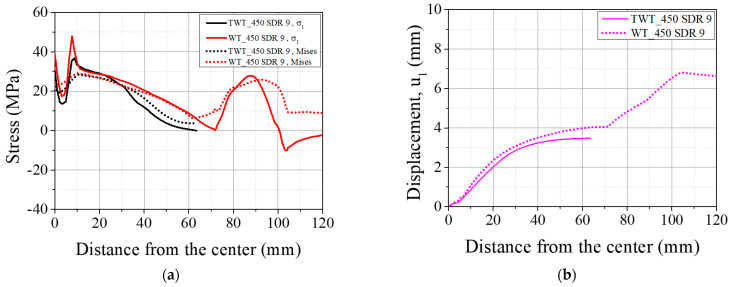
FEA results of Type B, 450 SDR 9 TWT and WT specimens at *F_max_*: (**a**) $\sigma_{v}$ and $\sigma_{1}$; (**b**) $u_{1}$.

Figure 14 illustrate the contours of von Mises stress ($\sigma_{v})$, axial displacement ($u_{1}$), and transverse deflection ($u_{3}$) at the maximum reaction force, *F_max_*. The axial stress ($\sigma_{1}),\;\;\sigma_{v}$ and $u_{1}$ of 110 SDR 9, 225 SDR 11, 315 SDR 11, and 450 SDR 9 TWT and WT specimens at *F_max_* are also shown in Figure 15, Figure 16, Figure 17 and Figure 18, respectively. Table 8 and Table 9 provide selected $\sigma_{v},$ $\sigma_{1,}$ $u_{1}$, and $u_{3}$ values at different locations along the specimen axis, as shown in Figure 12.

Typical behavior of $\sigma_{v}$ and $\sigma_{1}$ of WT specimens (Figure 15, Figure 16, Figure 17 and Figure 18 and Table 8) shows higher values at the traction hole (Position WP4 in Figure 12c), BF beadroll contact (Position WP2), and at the center of the BF bead (Position WP1). The magnitude of $\sigma_{v},$ and $\sigma_{1}$ depends on the Type and size of the WT specimen. On the other hand, $u_{1}$ is always the highest at the traction hole upper end (WP5). Similarly, as shown in the same figures and Table 9, in TWT specimens, $\sigma_{v}$ and $\sigma_{1}$ are always larger at the BF beadroll contact (Position NP2 in Figure 12c) than at the BF bead center (Position NP1), independent of the specimen type and size. In addition, $\sigma_{v}$ and $\sigma_{1}$ continue to decrease and $u_{1}$ increases towards the specimen end without interruption (Figure 15, Figure 16, Figure 17 and Figure 18). Furthermore, $u_{1}$ at NP2 and NP3 increases with the specimen size (Table 9).

Comparing 110 SDR 9 and 225 SDR 11 WT specimens (Figure 15a and Figure 16a, and Table 8), the maximum $\sigma_{1}$ at the traction hole (WP4) decreases by 9.06 MPa while increasing at the BF beadroll contact (WP2) by 2.25 MPa, going from 110 SDR 9 to 225 SDR11 size. Therefore, in 225 SDR 9 specimen while $\sigma_{v}$ is somewhat maintained approximately the same at WP4 (29.85 MPa) and WP2 (29.42 MPa) locations, the $\sigma_{1}$ is now the largest at WP2 (36.13 MPa) compared to 29.45 MPa at WP4. The location of the maximum $u_{1}$ is maintained at WP5; however, it was lowered from 7.61 mm (110 SDR) to 6.34 mm (225 SDR 11), as shown in Figure 15b and Figure 16b, and Table 8). The increase of $\sigma_{v}$ and $\sigma_{1}$ at the BF beadroll (WP2) and decrease at the traction hole (WP4) when going from 110 SDR 9 to 225 SDR 11 size is due to the increased width (60–80 mm) and the thickness (12.0–20.5 mm) of the unreduced portion of the specimen containing the traction hole.

In Type A TWT specimens (110 SDR 9 and 225 SDR 11), $\sigma_{v},$ $\sigma_{1}$, and $u_{1}$ distribution is less complex than the WT specimen (Figure 15a and Figure 16a, and Table 9). $\sigma_{v},$ and $\sigma_{1,}$ continue to decrease, and $u_{1}$ increases from NP2 to the specimen end as shown. The highest $\sigma_{v}$ and $\sigma_{1}$ are always at the BF beadroll contact (NP2), and the BF bead center (NP1) has lower values (Table 9). $\sigma_{v}$ and $\sigma_{1}$ at NP2 increase by 2.81 MPa and 5.98 MPa, going from 110 SDR 9 to 225 SDR size, and is attributed to the thickness increase (no width increase for the TWT specimen-Figure 3).

For Type B WT specimens (Figure 17a and Figure 18a, and Table 8), $\sigma_{v},$ and $\sigma_{1}$ at the traction hole (WP4) were always less than those values at the BF beadroll contact (WP2). $\sigma_{v}$ was 10.3% and 8.2% lower, and $\sigma_{1}$ was 19.8% and 40.6% lower for 315 SDR 11 and 450 SDR 9 models, respectively. On the other hand, the $u_{1}$ was always higher at WP5. The lower stresses at WP4 were due to the wider width of the unreduced portion of the specimen (100 mm) containing the traction hole and the increased thickness of the specimen (see Figure 3). With Type B TWT specimens, $\sigma_{v}$ and $\sigma_{1}$ were kept the largest at the BF beadroll contact (NP2) for all sizes as shown in Figure 17a and Figure 18a and Table 9.

In Type A specimens, $\sigma_{v}$, $\sigma_{1}$, and $u_{1}$ are seen to be lower in WT than TWT specimen in length between the region just after the BF beadroll contact (<10 mm) to the beginning of the traction hole (35 mm), as shown in Figure 15 and Figure 16. This is expected due to the shorter distance from the BF beadroll (WP2) to the free surface of the traction hole (WP3), giving rise to a higher stress gradient (faster drop) than in the longer Type B TWT specimen. In addition, the larger taper of the TWT (30°) waist compared to WT (0°) has caused a higher $u_{1}$ in the TWT specimen in the same region, as shown in Figure 15b and Figure 16b.

In Type B specimens, $\sigma_{v},$ $\sigma_{1}$, and $u_{1}$ are higher in the WT than the TWT specimen in length between the region around the BF beadroll (~20 mm) to the beginning of the traction hole at 70 mm Figure 17 and Figure 18). $\sigma_{v},$ $\sigma_{1}$, and $u_{1}$ are higher because the stress gradient is lower and $u_{1}$ higher due to the length of this region being longer than the total length of the TWT specimen and WT having a longer parallel side length (20 versus 25 mm).

For BF beadroll contacts (WP2 and NP2), $\sigma_{v}$ is similar between WT and TWT specimens (Figure 15, Figure 16, Figure 17 and Figure 18) for all sizes. That is, similar load behavior up to the point of onset necking (*F_max_*) is expected between each size WT and TWT specimens. The experimental force–displacement curves shown in Section 3.3.1 demonstrate that similar *F_max_* values are reached on each size of WT and TWT specimens. However, the displacements at *F_max_* are shown to be different due to the traction hole deformation in WT specimens.

Therefore, from the $\sigma_{1}$ and $u_{1}$ behavior, while a larger portion of the displacement in the Type A WT specimen arises from the traction hole deformation (84% for 110 SDR 11 size), the percentage decreases to 42% in the Type B specimen. However, with the TWT specimen, the $u_{1}$ comes from the specimen displacement, independent of the specimen type. Since the rate of $u_{1}$ increase is higher from the bead center to the taper end portion of the specimen (Figure 15, Figure 16, Figure 17 and Figure 18), a good part of the specimen deformation is expected to come from this portion of the TWT specimen.

**Figure 19:**
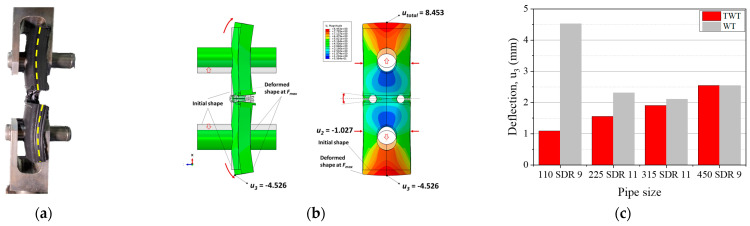
Transverse deflection of 110 SDR 9 WT specimen: (**a**) Experimental; (**b**) FEA result at *F_max_* (unit: mm); (**c**) Comparison of $u_{3}$ between TWT and WT specimens at *F_max_*.

The transverse deflection ($u_{3})$ of the 110 SDR 9 WT specimen is shown in Figure 19. $u_{3}$ causes the neutral plane of the specimen to bend during the tensile test and is the largest with the 110 SDR 11 size (Position WP5 in Table 8). When the size is increased to 225 SDR 11, $u_{3}$ is seen to decrease by about 50% and approximately maintained in larger specimens, as shown in Figure 19. On the other hand, with the TWT specimen, $u_{3}$ is smallest with the 110 SDR 9 size and increases incrementally as the size gets larger.

**Figure 20:**
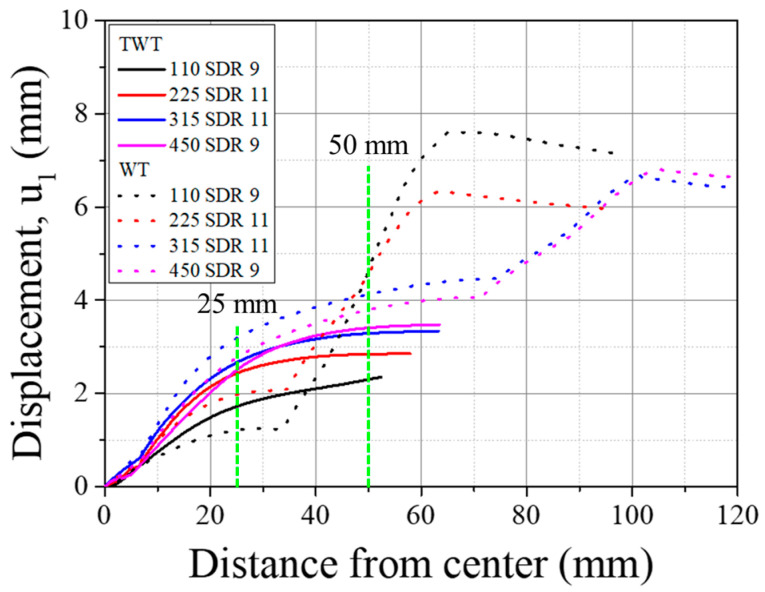
Comparison of $u_{1}$ along the specimen centerline (see Figure 12c).

**Figure 21:**
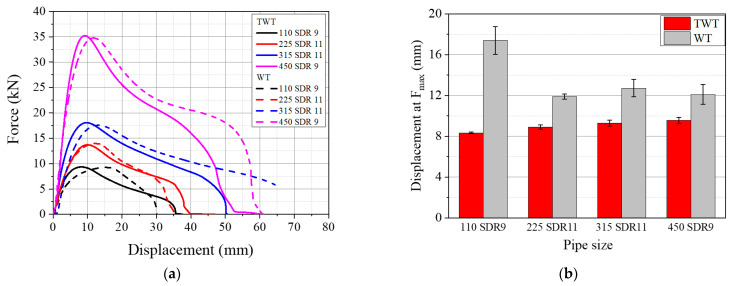
Comparison between TWT and WT specimens: (**a**) Load–displacement curves; (**b**) Onset necking displacement.

By comparing Figure 19 ($u_{3})$ with Figure 20 ($u_{1})$, one can observe that the excessive transverse deflection in 110 SDR 9 WT specimen brings about large specimen displacement. This is speculated to be due to the lower stiffness of the thinner 110 SDR 9 size compared to other larger sizes. However, with the TWT specimen, such large transverse deflection is not observed in 110 SDR 9 size, which indicates the stability of the specimen under the tensile test. As the specimen gets larger, the difference in $u_{3}$ (Figure 19) and $u_{1}$ (Figure 20) between TWT and WT gets smaller, indicating that a comparable load–displacement behavior to *F_max_* can be expected to occur. This is experimentally shown in Figure 21b, where the difference in displacement at *F_max_* becomes smaller with larger specimen sizes.

It is noted that based on the FE analysis, the 20 mm gauge length was selected for TWT and WT tensile tests (Figure 2). As shown in Figure 20, the change in displacement between the TWT and WT specimens is less than 5% for all specimen sizes when using a 20 mm (±10 mm) gauge length. Therefore, as expected, the experimental load–displacement curves measured with the extensometer are practically identical between the TWT and WT specimens at displacements up to 10 mm for the 110 SDR 9 size and up to 20 mm for the larger size, as presented in 3.3.2. In addition, the EN 12814-7 standard requirement to use an extensometer with a 50 mm (±25 mm) gauge length for WT specimens to avoid traction hole displacement is not required with TWT specimens. This is due to a small difference (less than 1.0 mm) in displacement measured at the specimen length 25 mm and at the specimen end from the BF bead center for all specimens, as shown in Figure 20.

### 3.3. Comparison of TWT and WT Specimens

#### 3.3.1. Load–Displacement Behavior Based on Crosshead Displacement

The tensile performance of TWT specimens with the shape and dimension (Table 1) proposed is compared to the WT specimen (ISO 13953) at 5 mm/min crosshead speed, as shown in Figure 21. The shape of the force–displacement curve is similar between the same size and Type TWT and WT specimens. However, some differences were observed in the total and onset necking (*F_max_*) displacements. With 110 SDR 9 size specimens, the displacement at *F_max_* of the WT specimen was ~100% larger than the TWT specimen. For sizes larger than 225 SDR 11, the difference was about ~30%, as shown in Figure 21b. In addition, the onset necking displacement is shown to be the maximum with the 110 SDR 9 WT specimen and largely decreases to a relatively constant value with larger size WT specimens. The displacement continues to increase in smaller increments with the increasing size of the TWT specimen, as shown. Therefore, in all sizes, the onset necking displacement of the TWT specimen is smaller than that of the WT specimen. However, the total displacement varies depending on the specimen type (Figure 21). With Type A specimens (110 SDR 9 and 225 SDR 11), TWT elongation is higher. And lower when Type B (315 SDR 11, 450 SDR 9) are compared. The difference is explained later with the FE analysis, and it is due to the presence of a fusion zone notch (Type A) versus the parallel side (Type B) of the TWT and WT specimens.

#### 3.3.2. Load-Displacement Behavior Based on Gage Length of 20 mm

**Figure 22:**
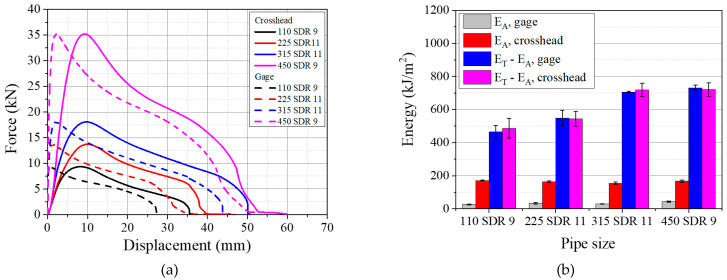
Extensometer versus crosshead measurements of TWT specimen; (**a**) Load–displacement curves; (**b**) Normalized energies calculated at *F_max_* ($E_{F_{max}})$ and the energy difference ($E_{Total}-E_{F_{max}}$). $E_{Total}$ is the normalized total fracture energy.

Per ISO 13953, the WT BF fracture energy is taken from the total area under the load–displacement curve based on the crosshead displacement. Thus, the WT or TWT BF fracture energy calculation based on the total area would grossly overestimate the fracture energy of the BF joint. Therefore, to better estimate the load–displacement behavior of the BF joint under the tensile load, measurement was carried out using a 20 mm gage length extensometer with the BF bead at the center (Figure 2). The result of the load–displacement curve using the extensometer is shown in Figure 22a for the TWT specimen, along with the result obtained using the crosshead displacement. In all sizes, the onset-necking load (*F_max_*) and the shape of the post-onset necking load–displacement behavior are similar between the extensometer and the crosshead measurements. However, the displacement at *F_max_* is much reduced with the extensometer measurement. Furthermore, this difference in displacement (crosshead versus extensometer) is seen to be maintained throughout the post-onset necking to failure, indicating that the post-onset necking displacement is mostly from the gage length region. Therefore, the pre-onset necking displacement of the crosshead measurement is mostly from the total length of the specimen, and the difference to the extensometer measurement becomes maximum at the onset necking load, as indicated in Figure 22a. These behaviors are due to the necking initiating and propagating from the point of contact between the BF beadroll and the pipe, where the stress concentration is created, as shown in Figure 15, Figure 16, Figure 17 and Figure 18.

In support of this, deformation energies from the load–displacement curves of the crosshead versus extensometer are compared in Figure 22b. $E_{F_{max}}$ is the energy normalized by the cross-sectional area of the TWT specimen, calculated at *F_max_* and $E_{Total}$ is the area normalized total energy to failure. The energy of pre-onset necking ($E_{F_{max}}$) from crosshead displacement is about five times larger than the energy from extensometer displacement for all TWT specimens. In comparison, the mean post-necking energies are practically the same (within 1~4.5% difference). Hence, confirming the source of pre and post-onset necking displacements as indicated above.

**Figure 23:**
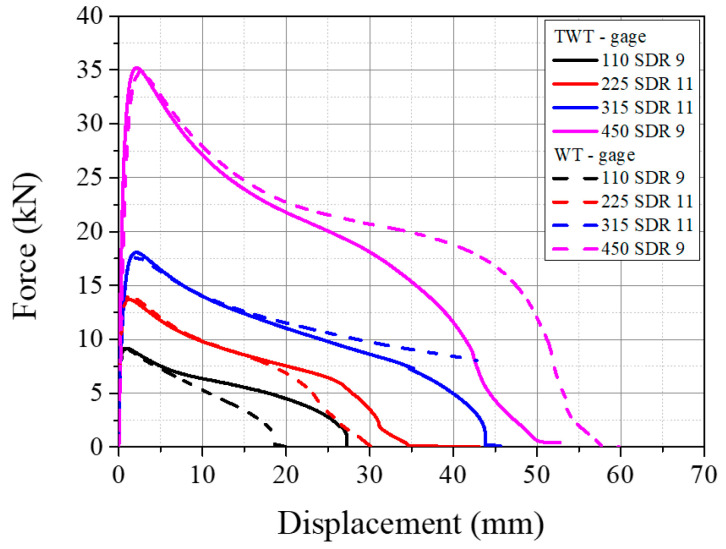
Load–displacement curves of TWT and WT specimens using a 20 mm gage length extensometer.

A comparison of load–displacement between the TWT and WT specimens is shown in Figure 23. They are practically identical up to the point of onset necking. And similar to the crosshead load–displacement behavior, the total displacement measured is dependent on the specimen type. With Type A specimens (110 SDR 9 and 225 SDR 11), the TWT displacements are larger than the WT displacements and smaller when Type B (315 SDR 11, 450 SDR 9) are compared. The deviation in the load–displacement curve after the onset necking is seen to occur at the smallest displacement for 110 SDR 9 (TWT-20 mm notch versus WT-10 mm diameter notch), followed by 225 SDR 11 (both are 20 mm diameter notch). For Type B specimens, TWT (20 mm parallel side) and WT (25 mm parallel side) showed more or less the same behavior throughout the necking region up to a 20 mm displacement.

Therefore, the TWT specimen shape and dimension proposed in Table 1 gives practically the same load–displacement of the WT specimen in the BF joint area up and beyond the onset necking. On the other hand, the crosshead displacement at the maximum load is smaller with the TWT specimen. Hence, indicating that two specimens provide the same deformation behavior at the BF joint area. At the same time, the TWT specimen provides a better estimate of the BF fracture energy when determined from a more convenient crosshead load–displacement curve. In addition, since the TWT specimens are smaller than the WT specimens, the BF area to be tested is about 60% larger (Table 3). Furthermore, the TWT specimen preparation is made simpler by removing tractions holes and having the width of the unreduced part of the specimen fixed for all pipe-size BF joints (Figure 3). Thus, a single-size surface angle grip is made to accommodate all-size TWT specimens (Figure 5). In terms of the practicality of testing, TWT specimens are more stable (push-in grips) (Figure 2a, Figure 5 and Figure 11a) than WT specimens (pin rods) (Figure 2b and Figure 11b).

## 4. Conclusions

The tapered waist tensile (TWT) test was developed and applied to evaluate the integrity of high-density polyethylene pipe butt fusion (BF) joints. Experimental and numerical analyses of the TWT specimens were performed to obtain an optimum design for the BF joint destructive test. As a result, the Type A TWT specimen has been shown to be suitable for pipe thicknesses of 25 mm and less, and Type B has been suggested for thicknesses greater than 25 mm. Type A TWT specimens were characterized by a taper angle to a circular notch in the fusion zone, whereas Type B was designed with the same taper angle to a parallel sided BF region. In addition to the taper angle created to support the tensile loading, other important features of the TWT specimen include a 20 mm diameter notch (or 20 mm parallel side length) in the BF area, a 60 mm unreduced width, and a 25 mm reduced fusion zone width. The same dimensions apply to all pipe sizes and standard dimensional ratios (SDR). Furthermore, no traction holes are used for loading the specimen. The use of simple push-in taper-support grips with an angle of 30° was made to minimize specimen contribution in calculating the BF fracture energy. Depending on the test time requirement, the tensile test speeds of 5 mm/min and 10 mm/min can be used.

These features separate the TWT specimens from the WT specimens and reduce specimen displacement by more than 100% (for the 110 SDR 9 size) and 30% (225 SDR 11 and larger) at the onset of necking load. Similarly, the stability of the TWT specimens during tensile testing is significantly better than the WT specimens, as analyzed by the development of lower transverse deflections. Additionally, TWT specimens can test at least 60% more portions of pipe BF joints than WT specimens. Therefore, compared to ISO 13953 waisted tensile (WT) specimens, TWT specimens offer significant improvements in BF joint tensile testing in terms of simplicity, adaptability, stability, and accuracy in specimen preparation, testing, and analysis. At the same time, the TWT specimens exhibited BF fracture behavior equivalent to that of the WT specimens, as evidenced by similar load–displacement curves obtained using a 20 mm gauge length extensometer centered on the BF joint.

Finally, the proposed TWT specimen will be applied to evaluate the various flawed high-density polyethylene BF joints. In addition, it can be used as an alternative to the WT specimen of ISO 13953.

## Figures and Tables

**Table 1:** Dimensions of TWT specimens.

Symbol (See Figure 3a,b)	Description (mm)	Type A (*t* < 25 mm)	Type B (*t* ≥ 25 mm)
*A*	Overall length	100	120
*B*	Width at ends	60	
*C*	Length of narrow parallel sided portion	NA	20
*D*	Width of narrow portion	25	
*R*	Tapered waist fusion zone notch radius	10	
*Φ*	Tapered (Grip) support angle	30°	

**Table 2:** Dimensions of WT specimens per ISO 13953.

Symbol (See Figure 3c–e)	Description (mm)	Type A (*t* < 25 mm)		Type B (*t* ≥ 25 mm)
		*d_n_* ≤ 160	*d_n_* > 160	
*A*	Overall length	180		250
*B*	Width at ends	60	80	100
*C*	Length of narrow parallel sided portion	NA		25
*D*	Width of narrow portion	25		
*R*	Radius	5	10	25
*G*	Initial distance between grips	90		165
*I*	Diameter of the traction holes	20 ± 5		30 ± 5

**Table 3:** Percent BF area per pipe covered by TWT and WT specimens.

Pipe Size	Number of Samples from a Whole Pipe		BF Area Evaluated (%)	
	TWT	WT	TWT	WT
110 SDR 9	4	4	32.6	32.6
225 SDR 11	8	5	31.1	19.5
315 SDR 11	12	7	33.4	19.5
450 SDR 9	18	10	35.8	19.9
914 SDR 11	39	23	37.4	22.0

**Table 4:** Maximum load and corresponding displacement of Type A, 110 SDR 9 TWT, and WT specimens.

Specimen Notch Radius (mm)	*F_max_* (kN)	Displacement @ *F_max_* (mm)
TWT_5	8.97 ± 0.14	8.1 ± 0.2
TWT_10	9.08 ± 0.13	8.3 ± 0.1
WT_5	8.79 ± 0.31	17.4 ± 1.4

**Table 5:** Maximum load and corresponding displacement of Type A, 225 SDR 11 TWT, and WT specimens.

Specimen Notch Radius (mm)	*F_max_* (kN)	Displacement @ *F_max_* (mm)
TWT_5	14.28 ± 0.39	9.0 ± 0.2
TWT_10	13.70 ± 0.12	8.9 ± 0.2
WT_10	13.77 ± 0.31	12.4 ± 0.6

**Table 6:** Maximum load and corresponding displacement of Type B, 315 SDR 11 TWT, and WT specimens.

Specimen Parallel Side (mm)	*F_max_* (kN)	Displacement @ *F_max_* (mm)
TWT_0	20.11 ± 0.12	9.9 ± 0.4
TWT_10	18.65 ± 0.12	9.6 ± 0.8
TWT_20	18.09 ± 0.15	9.6 ± 0.1
WT_25	17.56 ± 0.04	12.7 ± 0.9

**Table 7:** Maximum load and corresponding displacement of Type B, 450 SDR 9 TWT, and WT specimens.

Specimen-Parallel Side (mm)	*F_max_* (kN)	Displacement @ *F_max_* (mm)
TWT_10	37.37 ± 0.40	8.8 ± 0.6
TWT_20	35.13 ± 0.09	9.6 ± 0.3
WT_25	34.50 ± 0.87	12.1 ± 1.0

**Table 8:** $\sigma_{v},$ $\sigma_{1,}$ $u_{1}$, and $u_{3}$ values at selected positions (Figure 12c) along the longitudinal direction from the center of the BF joint to the tip of the WT specimens at *F_max_*.

WT Specimen	Position WP1			Position WP2				Position WP4		Position (WP5-WP6)	
	$\sigma_{v}\;(\mathrm{MPa})$	$\sigma_{1}\;(\mathrm{MPa})$	${\left|u_{3}\right|}_{max}$ (mm)	$\sigma_{v}\;(\mathrm{MPa})$	$\sigma_{1}\;(\mathrm{MPa})$	${\left|u_{1}\right|}_{max}$ (mm)	${\left|u_{3}\right|}_{max}$ (mm)	$\sigma_{v}\;(\mathrm{MPa})$	$\sigma_{1}\;(\mathrm{MPa})$	${\left|u_{1}\right|}_{max}$ (mm)	${\left|u_{3}\right|}_{max}$ (mm)
110 SDR 9	22.51	23.44	3.08	26.56	33.88	1.12	3.07	30.81	38.51	7.61	4.53
225 SDR 11	23.68	46.90	2.37	29.42	36.13	1.50	2.40	29.85	29.45	6.34	1.20
315 SDR 11	26.84	45.49	2.09	29.84	41.30	3.79	2.11	26.78	33.14	6.67	2.11
450 SDR 11	23.87	39.70	2.50	29.16	47.88	3.47	2.55	26.78	28.45	6.81	2.55

**Table 9:** $\sigma_{v},$ $\sigma_{1,}$ $u_{1}$, and $u_{3}$ values at selected positions (Figure 12c) along the longitudinal direction from the center of the BF joint to the tip of the TWT specimens at *F_max_*.

TWT Specimen	Position NP1			Position NP2				Position NP3	
	$\sigma_{v}\;(\mathrm{MPa})$	$\sigma_{1}\;(\mathrm{MPa})$	${\left|u_{3}\right|}_{max}\;(\mathrm{mm})$	$\sigma_{v}\;(\mathrm{MPa})$	$\sigma_{1}\;(\mathrm{MPa})$	${\left|u_{1}\right|}_{max}\;(\mathrm{mm})$	${\left|u_{3}\right|}_{max}\;(\mathrm{mm})$	${\left|u_{1}\right|}_{max}\;(\mathrm{mm})$	${\left|u_{3}\right|}_{max}\;(\mathrm{mm})$
110 SDR 9	21.44	29.67	1.09	26.60	34.05	1.52	1.08	2.39	0.51
225 SDR 11	24.06	30.23	1.56	29.41	40.03	2.63	1.52	2.85	0.51
315 SDR 11	25.99	33.77	1.88	29.26	34.91	3.12	1.91	3.34	0.51
450 SDR 11	22.34	32.28	2.51	28.00	38.62	3.27	2.55	3.47	0.25

## Data Availability

The data that supports the findings of this study are available within the article.
